# The *panniculus carnosus* muscle: A novel model of striated muscle regeneration that exhibits sex differences in the *mdx* mouse

**DOI:** 10.1038/s41598-019-52071-2

**Published:** 2019-11-04

**Authors:** Ola A. Bahri, Neia Naldaiz-Gastesi, Donna C. Kennedy, Antony M. Wheatley, Ander Izeta, Karl J. A. McCullagh

**Affiliations:** 1 Department of Physiology, School of Medicine, Human Biology Building, National University of Ireland Galway, Galway, H91 W5P7 Ireland; 2 Regenerative Medicine Institute, School of Medicine, National University of Ireland Galway, Galway, Ireland; 3 Biodonostia Health Research Institute, San Sebastian, Spain

**Keywords:** Muscle stem cells, Skeletal muscle, Neuromuscular disease

## Abstract

The dermal striated muscle *panniculus carnosus* (PC), prevalent in lower mammals with remnants in humans, is highly regenerative, and whose function is purported to be linked to defence and shivering thermogenesis. Given the heterogeneity of responses of different muscles to disease, we set out to characterize the PC in wild-type and muscular dystrophic *mdx* mice. The mouse PC contained mainly fast-twitch type IIB myofibers showing body wide distribution. The PC exemplified heterogeneity in myofiber sizes and a prevalence of central nucleated fibres (CNFs), hallmarks of regeneration, in wild-type and *mdx* muscles, which increased with age. PC myofibers were hypertrophic in *mdx* compared to wild-type mice. Sexual dimorphism was apparent with a two-fold increase in CNFs in PC from male versus female *mdx* mice. To evaluate myogenic potential, PC muscle progenitors were isolated from 8-week old wild-type and *mdx* mice, grown and differentiated for 7-days. Myogenic profiling of PC-derived myocytes suggested that male *mdx* satellite cells (SCs) were more myogenic than female counterparts, independent of SC density in PC muscles. Muscle regenerative differences in the PC were associated with alterations in expression of calcium handling regulatory proteins. These studies highlight unique aspects of the PC muscle and its potential as a model to study mechanisms of striated muscle regeneration in health and disease.

## Introduction

Duchenne muscular dystrophy (DMD) is the most common lethal congenital children’s disorder^1,2^. DMD is due to a mutation in the gene encoding the sarcolemma-linked dystrophin protein, resulting in its absence. Dystrophin is associated with other proteins as the dystrophin glycoprotein complex (DGC) and provides mechanical stability and signalling functions to the muscle cell^1^. The absence of dystrophin results in a loss of muscle integrity and sensitivity to mechanical stress^3^. The loss in structural stability and signalling that the DGC provides results in multiple cycles of degeneration-regeneration with the onset of fibrosis, necrosis, followed by collagen and fat deposition. Muscle specific stem cells actively regenerate the defective muscle fibres, until they become exhausted and can no longer sustain the regenerative process^4–7^. In addition, the ruptured sarcolemma results in extracellular calcium (Ca^2+^) influx and Ca^2+^ leakage from the sarcoplasmic reticulum (SR) into the sarcoplasm, both of which lead to elevations in intracellular Ca^2+^ levels that contribute to the dystrophic phenotype^8,9^.

Muscle stem cells are required for regeneration and for normal growth of muscle^10^. The canonical muscle stem cells are satellite cells (SCs), which reside between the basal lamina and the sarcolemma of the adult muscle fibre. In adult muscle, SCs are mitotically quiescent and are identifiable by specific expression of paired-box transcription factor Pax7^11^. Upon injury or disease, SCs are activated to become muscle precursor cells which fuse with degenerating myofibers or each other to generate new myofibers, which are small and have centrally located nuclei^12^. Muscle regeneration is coordinated by the expression of myogenic regulatory factors (MRFs) including MyoD, Myf-5, MRF4 and myogenin. In DMD, the SCs become exhausted in their ability to sustain the high demand for muscle regeneration, leading to complete muscle impairment^5,13^. Muscle SCs are known to depend on intrinsic and extrinsic factors^6^. Sex influences SC activity, which is thought to be mediated by sex hormone levels and/or intrinsic sex differences, but the precise mechanisms remain unclear^14,15^.

Skeletal muscle fibres are heterogeneous in cellular make-up indicated by the myosin heavy chain (MyHC) composition, in which there are four types: slow twitch type I, and fast twitch types IIA, IIX and IIB (IIX in humans)^16^. In DMD, both in human and *mdx* mice, the fast twitch IIX and IIB fibre types respectively, are more susceptible to degeneration than the slow type I fibres^17–19^. In addition, some muscle groups with different anatomical locations and functions, show heterogeneity in their physiological function and response to disease^20^. The extraocular muscles of the eye, the laryngeal and masticatory muscles are resistant to degeneration in DMD, while tongue muscles are mildly affected. In contrast, the more commonly described limb muscles degenerate with greater frequency in DMD^20^. These dissimilarities in susceptibility to muscle degeneration have in part been attributed to intrinsic differences including superior calcium homeostasis due to higher levels of calcium buffering/regulatory proteins in the degenerative resistant muscles^21–23^.

Among the many striated muscles in the body, the *Panniculus Carnosus* (PC) muscle shows unique regenerative characteristics, but it has not been extensively studied. The PC striated muscle is located within the subcutaneous layer of the skin. While the PC is vestigial in humans, it is widely present in the dermis of quadrupeds including rodents^24^. The PC has been studied at the anatomical level in different mammalian species^24^. However, PC muscle at the cellular, subcellular and molecular levels has been poorly defined. One study serendipitously discovered that the PC muscle in healthy mice exhibits a relatively high turnover compared to limb muscles, in the absence of any injury^25^. Uniquely, exogenous bone marrow-derived cell engraftment into the PC muscle was many times greater than for any other muscle in the mouse^25^. Recently, Garcia-Parra et al re-investigated the PC as a potential candidate for muscle/dermal bio-engineering applications^26,27^. Naldaiz-Gastesi et al went as far to delineate the origin of the resident PC muscle stem cells as being from the canonical Pax7 specified satellite cells and not to some non-canonical multipotent tissue resident progenitor cell as previously thought^28^.

Herein, we investigate the PC striated muscle, at the cellular and molecular levels in healthy mice and in the *mdx* mouse model of DMD. The first aim was to study the morphology, and regeneration of the dorsal PC from wild-type and *mdx* male mice at age 6 weeks and 12 weeks. The mouse ages were chosen as these span the period of heightened degeneration-regeneration cycles^29–31^. A second aim was to examine the whole-body distribution of PC in wild-type and *mdx* male mice at 1-year of age, a time point of severe fibrosis^32^. A third aim, was to analyse the effect of sex on the PC muscle regeneration *in vivo*. Finally, to gain a closer cellular insight into the regenerative activity of the PC, we isolated satellite cells (SCs) of the PC from male and female wild-type and *mdx* mice and assessed their myogenic activity *in vitro*. SC density and calcium regulatory genes were assessed in the PC muscles. The findings from this study provide a better understanding of the PC muscle in a diseased state and identified PC regeneration to be sex-related. We propose that the characteristics of PC muscle in normal and *mdx* mice, make the PC muscle an attractive model for studying mechanisms of muscle regeneration in healthy and diseased states.

## Results

### Muscle fibre types in PC of wild-type and dystrophic mdx mice

**Figure 1:**
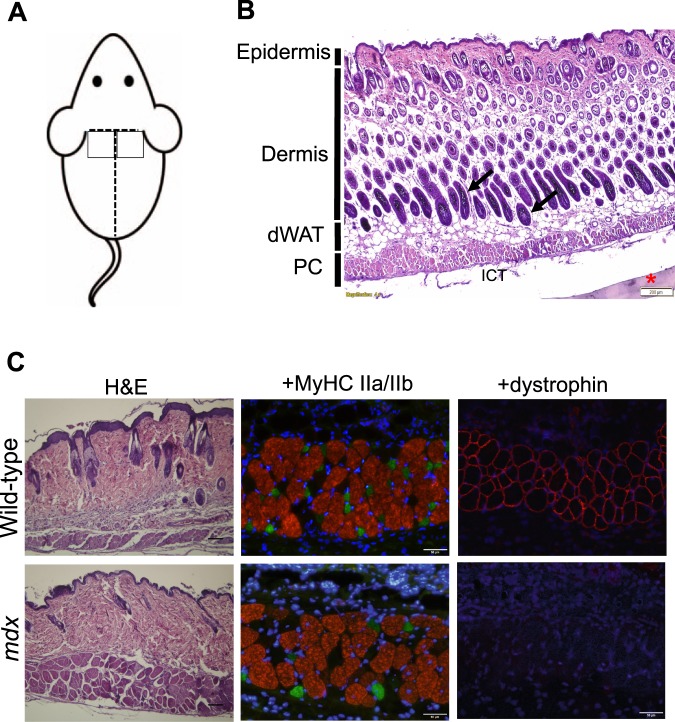
Panniculus Carnosus (PC) muscle consists of fast twitch type IIB glycolytic fibres in both wild type and *mdx* male mice. (**A**) Illustration of the two areas of the superior-mid dorsal skin of the mouse used for analyses. Two skin fragments of each 1 cm^2^ in dimension were harvested from the dorsum of a 6-week old male mouse. **(B)** Representative morphology of the murine panniculus carnosus (PC) muscle. Haematoxylin & Eosin (H&E) stained transverse section of paraffin embedded skin tissue from the superior-mid dorsum of a 6-week old male *mdx* mouse. The PC is a 3–5 myofiber wide striated muscle layer, located beneath the dermal white adipose tissue (dWAT). Arrows indicate hair follicles. ICT: Interstitial connective tissue; *PVDF membrane; Scale bar = 200 µm. **(C)** Sample of paraffin-preserved mid dorsal skin cross-section histochemically stained with H&E demonstrating the anatomical location of the PC muscle in 8-week old male wild-type and *mdx* skin tissues. Scale bar is 200 µm. PC unfixed frozen cross-sections from 8-week old male wild-type and *mdx* mice, immunofluorescently stained with anti-myosin IIA (green), anti-myosin IIB antibodies (red) and nuclei stained with DAPI (blue). Scale bar = 50 µm. Immunofluorescent staining for dystrophin confirms the *mdx* genotype. Positively stained fibres for dystrophin in 8-week old male wild-type mid-dorsal PC striated muscle in contrast to *mdx* fibres, which are null for dystrophin. Nuclei stained with DAPI (blue). Scale bar = 50 µm.

Murine skin is composed of multiple layers from the outer epidermis, dermis, panniculus adiposus (PA) and a thin 3–5 myofiber wide (~100 μm) striated muscle layer, located between the PA and interstitial connective tissue (ICT) layers on the dorsum of mice called the panniculus carnosus (PC) (Fig. 1A). These same tissue layers visualised with haematoxylin and eosin (H&E) staining of transverse dorsal skin sections from both wild-type and *mdx* mice (Fig. 1C). The PC is reported to be composed of striated muscle fibres expressing fast type II MyHC in normal mice^25^. To delineate which subtypes of myosin expressing fibres are present in the PC in wild-type and *mdx* mice, we subjected muscle sections to a panel of MyHC isoform specific antibodies. The PC fast fibre phenotype was confirmed by cross-reaction with a pan anti-fast myosin antibody in both wild-type and *mdx* transverse dermal sections (data not shown). Furthermore, the PC had strongest cross-reaction with an anti-MyHC-IIB specific antibody indicating a prevalence of fast-glycolytic type IIB muscle fibres (Fig. 1C in red). A much lower frequency of muscle fibres immunopositive for an antibody against MyHC-IIA (Fig. 1C in green) was found, indicating the low presence of fast-oxidative-glycolytic type IIA fibres. There was no cross-reaction of fibres against an anti-slow myosin MyHC-I antibody (data not shown). The muscle genotype of mouse tissues was confirmed by incubating sections with an anti-dystrophin antibody, where wild-type PC tissues were immunopositive for dystrophin in the surrounding sarcolemma, and *mdx* PC muscle sections were negative for dystrophin (Fig. 1C).

### Whole-body wide distribution of PC muscle

**Figure 2:**
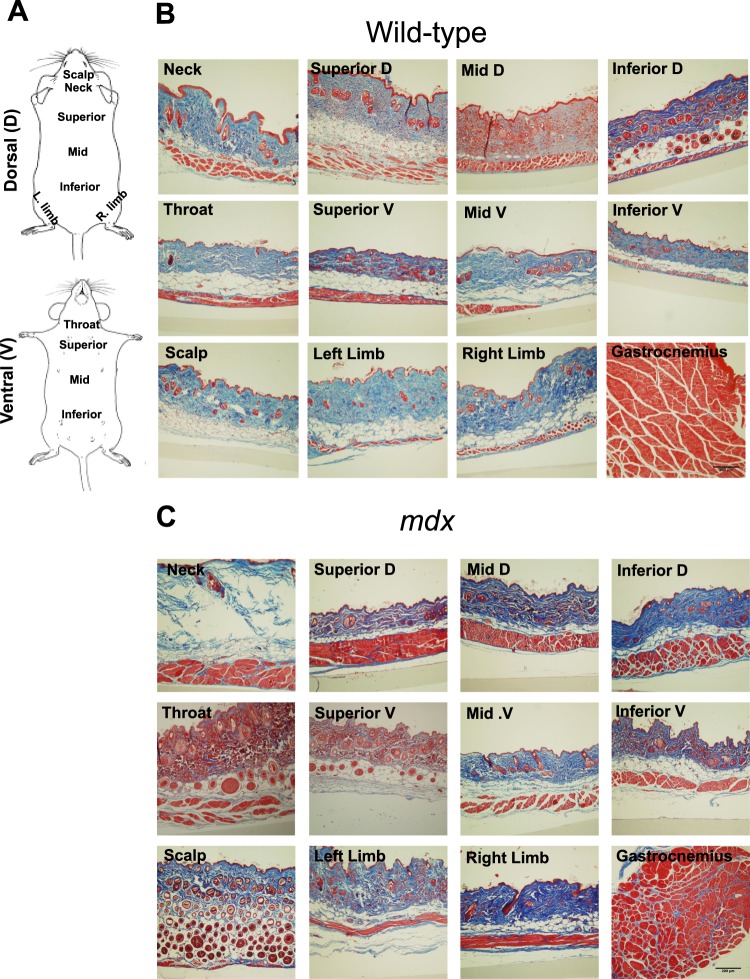
The PC muscle is widely distributed in wild type and *mdx* male mouse dermal tissues. (**A**) Schematic shows the locations of skin fragments examined on dorsal (**D**) and ventral (V) parts of both wild-type and *mdx* mice. Adapted from Treuting *et al*.^64^. **(B,C)** Representative histochemical Masson’s Trichrome blue stain of paraffin-preserved skin fragment cross-sections from 11 anatomical locations from 1-year old male wild-type and *mdx* mice. The outer epidermis layer appears red, dermis layer is blue, and the striated muscle PC layer is rich red. A gastrocnemius muscle cross-section is included as a control for skeletal muscle staining. Scale bar = 200 μm.

To understand the whole-body distribution of PC in the healthy mouse and the effect of *mdx* dystrophy on the diverse anatomical locations of PC, we harvested dermal fragments from eleven different locations around the whole body of 1-year old wild-type and *mdx* mice (Fig. 2A). Paraffin embedded fragments were sectioned and stained with Trichrome blue (Fig. 2B,C). A thin 3–5 myofiber (~100 μm) wide sub-dermal striated muscle layer consistent with the PC location was robustly stained rich red in all skin cross-sections examined (Fig. 2B,C). The gastrocnemius muscle was included as a control. The PC was widely distributed across almost all the different body skin fragments from both wild-type and *mdx* mice. Noticeably, the PC muscle layer was thicker (~200 μm) in dermal fragments from *mdx* compared to wild-type mice (Fig. 2B,C and Suppl. Fig. S2). Furthermore, the PC appeared to be thicker in dorsal compared to ventral sections and with a tendency to be wider in rostral compared to caudal sections in both wild-type and *mdx* mice.

### Regeneration in PC muscle from wild-type and mdx mice

**Figure 3:**
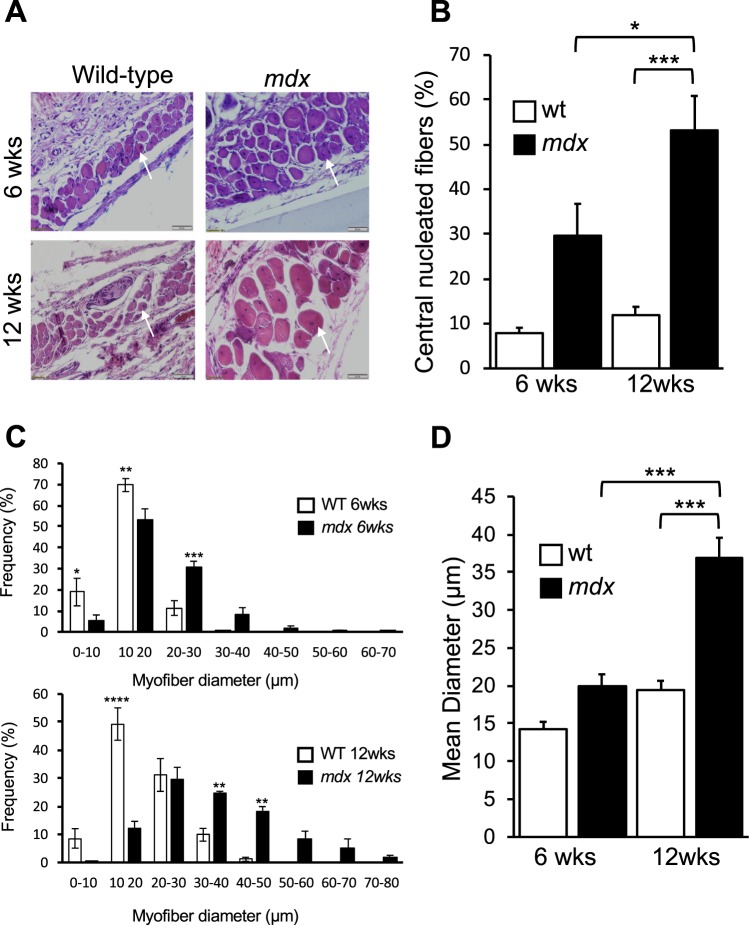
Highly regenerative PC muscle demonstrates dystrophic morphology and hypertrophy in *mdx* male mice. (**A**) Histological sections of PC muscle stained with H&E. Representative view of PC striated muscle demonstrates centrally nucleated fibre phenotype in wild type and *mdx* of paraffin skin tissue sections (superior-mid dorsal) from 6 and 12-week-old male mice. Arrows indicate CNFs **(B)** Quantitative comparison of the percentage of fibres with central nuclei in 6 and 12-week-old wild-type and *mdx* mice. CNFs were quantified from 4–5 non-overlapping fields per muscle section at 10x magnification. Data were analysed by 2-way ANOVA and Tukey’s multiple comparison test (*p < 0.05, ***p < 0.001; n = 4 mice). **(C)** Muscle fibre size distribution based on Feret’s diameter measurements on PC tissues from 6 and 12-week-old male wild-type and *mdx* mice. Fibre sizes were determined from 4–5 non-overlapping fields per muscle section at 20x magnification Data bin sizes were analysed by t-tests and are represented as means ± SEM; *p < 0.05,**p < 0.01,***p < 0.001 and ****p < 0.0001; n = 4 mice for each genotype. **(D)** Mean myofiber diameters in 6-week and 12-week old wild-type and *mdx* male mice. Data were analysed by 2-way ANOVA and Tukey’s multiple comparison post-hoc test. Data are represented as means + SEM; ***p < 0.01; n = 4 mice for each sex and genotype. Data are means + SEM with n = 4 mice.

**Figure 4:**
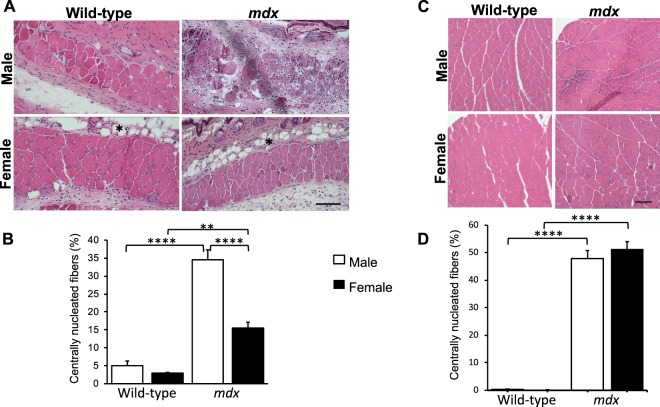
PC muscle degeneration/regeneration is sex-related. (**A**) Histological representative of cryogenically sectioned PC tissues (mid-dorsal) from 8-week old male and female wild-type and *mdx* mice (scale bar = 100 µm). ^*^Dermal white adipose tissue (dWAT) is indicated. **(B)** Quantitative comparison of the percentage of PC muscle fibres with central nuclei in male and female wild-type and *mdx* mice. **(C)** Histological representative of gastrocnemius cryo-sections from 8-week-old male and female wild-type and *mdx* mice. **(D)** Quantitative comparison of the percentage of gastrocnemius muscle fibres with central nuclei in 8-week-old male and female wild-type and *mdx* mice. Data were analysed by 2-way ANOVA and Tukey’s multiple comparison post-hoc test Data are represented as means + SEM; **p < 0.01, ****p < 0.0001; n = 4 mice for each sex and genotype.

To examine the regeneration levels and the dystrophic histopathology of the PC muscle, we scored myofibers with central nuclei in H & E stained sections at 6 and 12 weeks in both groups (Fig. 3A,B). The *mdx* fibres showed a greater percentage of CNFs compared to wild-type in both 6-wk and 12-wk old mice. A 4-fold higher CNF level was seen in young *mdx* mice (29.6 ± 7.1%) when compared to their aged matched counterparts (7.9 ± 1.2%). A further elevation was observed in older *mdx* mice with nearly a 5-fold (53 ± 7.7%) higher frequency of CNFs compared to matching wild-type mice (11.9 ± 1.8%). No significant difference was seen between the percent of centrally nucleated myofibers between the two ages in the control mice (Fig. 3B). As a comparison, CNFs in the gastrocnemius muscles of healthy mice are <1% (see below and Fig. 4D).

### Heterogeneity of PC muscle fibre size in wild-type and mdx

To further evaluate the effects of age and dystrophy on the PC muscle, we examined the homogeneity of the PC myofiber size. Dermal tissue sections were fluorescently stained with WGA-Alexa 488 to demarcate the membrane of each myofiber, to facilitate morphometric analysis (Suppl. Fig. S1). As a morphometric index, minimal Feret’s diameter was measured on stained PC transverse sections in 6-week and 12-week old wild-type and *mdx* mice respectively (Fig. 3C,D). The frequency distribution of myofiber diameters was shifted to larger myofibers in *mdx* compared to age-matched wild-type mice at both ages (Fig. 3C). The mean diameter at 6 weeks (19.9 ± 1.6 µm) was increased by 1.4-fold in *mdx* compared to its wild-type control (14.2 ± 1 µm) but this was not significant (Fig. 3D). In contrast, the mean diameter at 12 weeks was significantly increased by 1.9-fold in *mdx* PC (36.8 ± 2.7 µm) above its wild-type control (19.4 ± 1.2 µm) (Fig. 3D; p < 0.001). These morphometric indices increased with age from 6 to 12 weeks of age in the *md*x mouse (Fig. 3C,D; p < 0.001).

Noticeably, there was a variation in the thickness of the three dermal layers: dermis, dermal white adipose tissue (dWAT) and PC muscle, between the groups based on age and genotype (Suppl. Fig. S2). Wild-type and *mdx* 6-week-old mice had similar relative thickness for the three dermal skin layers. In 12-week-old *mdx* mice there were significant alterations in all dermal layers. The dermis was reduced by half in the mdx compared to wild-type mice (p < 0.01). The dWAT layer was increased 2-fold (p < 0.05) and the PC muscle was increased 2.3-fold (p < 0.001) in the 12-week mdx mice compared their wild-type counterparts. Dermal layers were also significantly different between 6 and 12 week *md*x mice with increases in dWAT (p < 0.01) and PC (p < 0.0001) layers, and a decrease in the dermis (p < 0.001).

### Sex-related differences in PC and limb muscle degeneration/regeneration in wild-type and mdx mice

To evaluate the effect of sex on the degenerative/regenerative behaviour, PC tissues from 8-week-old male and female wild-type and *mdx* mice were analysed for CNFs. In the male *mdx* 35% of PC muscle fibres had CNFs, which is 7-fold higher than the mean percentage recorded in normal male PC (p < 0.001). Furthermore, a sex effect was shown in *mdx* in which the percentage of CNFs in female *mdx* (15.5 ± 0.02%) was 2.3 times lower than the percentage shown in male *mdx* (Fig. 4A,B; p < 0.001). In contrast, no differences were found in gastrocnemius limb muscles of male and female, wild-type and *mdx* mice (Fig. 4C,D; p < 0.001).

### Myogenic differentiation of PC muscle satellite stem cells from wild-type and mdx male and female mice

To investigate the myogenic activity of PC in dystrophic mice and the effect of sex, PC derived satellite cells were isolated and cultured for 7 days from the dorsal skin of mice.

**Figure 5:**
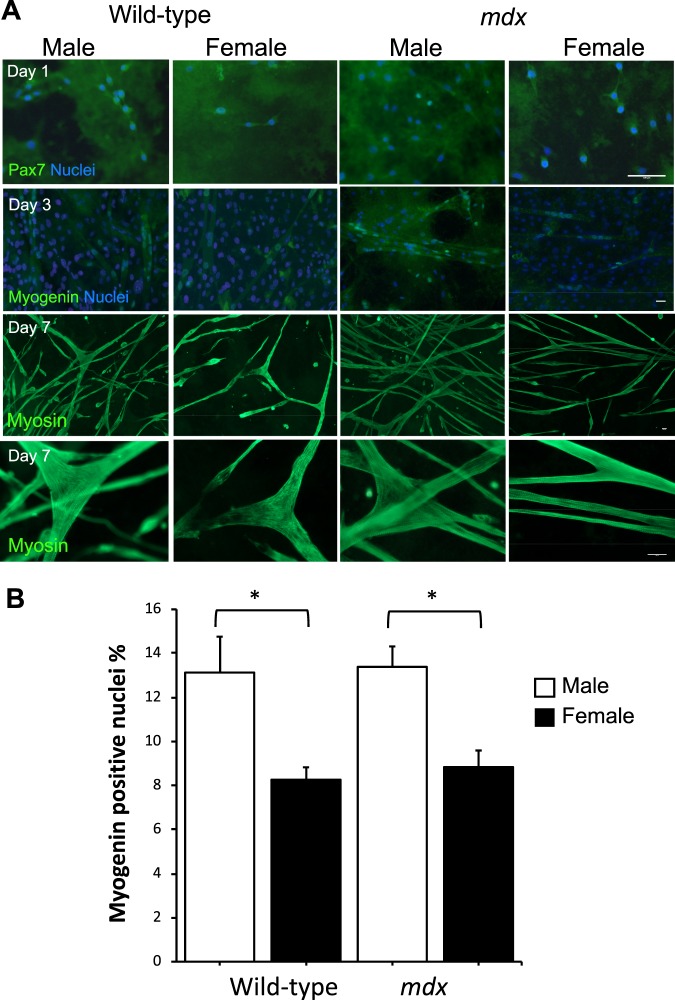
Comparison of *in vitro* myogenicity of PC derived cells from 8-week old wild-type and *mdx* mice of different sex. (**A)** Representative immunofluorescent detection of myogenic markers Pax7, myogenin and adult pan-myosin heavy chain (A4.1025) at day 1, 3 and 7, respectively. Pan-myosin staining in on day 7 is shown at two different power magnifications using 10x and 40x objective lens respectively. Scale bars = 100 µm. **(B)** Immunofluorescently stained cells for myogenin at day 3 of differentiation were scored for percentage of myogenin positive nuclei as an index of myoblast fusion and myogenicity. All cultures were seeded with similar cell number in each well. Data were analysed by 2-way ANOVA and Tukey’s multiple comparison post-hoc test. Data are represented as means + SEM; *p < 0.05; n = 4 mice.

Dermospheres started to cluster from day 2 (Suppl. Fig. S3). However, no obvious difference was noted between the dermosphere formation or expansion in wild-type and *mdx* cultures. Equal densities of progenitor cells were allowed to differentiate for 7 days in differentiation medium. Multi-nucleated myotubes were visualized by the third day and myofiber twitching was observed by the 5^th^–6^th^ day (Suppl. Movies S1). Myogenesis markers Pax7, Myog, MyHC2 (MyHC-2A) and MyHC3 (embryonic myosin) were examined at mRNA level on days 1, 3 and 7. Pax7 was detected on day 1 (Fig. 5A) and was still present on day 3 (data not shown). Myogenin was absent on day 1, detected on day 3 (Fig. 5A,B) and continued to be detectable on day 7 (data not shown). Myotubes readily expressed the adult MyHC by day 7 and showed the differentiated sarcomere organization (Fig. 5A). Immunofluorescence analysis revealed that while genotype had no influence on myogenin expression, sex had an effect both in wild-type and *mdx* mice. The mean percentage of myogenin positive nuclei in both female wild-type (8.27 ± 0.56%) and *mdx* (8.84 ± 0.76%) was significantly lower by 1.5-fold compared to their male counterparts, 13.13 ± 1.6% and 13.39 ± 0.9% respectively (Fig. 5B; p < 0.05).

**Figure 6:**
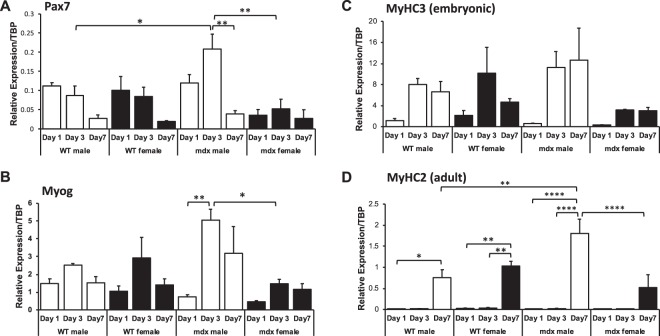
Comparison of *in vitro* myogenicity of PC derived cells from 8-week old wild-type and *mdx* mice of different sex by gene expression analysis. **(A**–**D)** Real-time quantitative PCR (RT-qPCR) of myogenic factors **(A)** Pax7, **(B)** Myogenin (Myog), **(C)** embryonic myosin (MyH3) and **(D)** adult myosin (MyH2) at day 1, 3 and 7 of differentiation in culture. Relative expression is target gene relative to the TATA binding protein (TBP). Data were analysed by 3-way ANOVA and Tukey’s multiple comparison post-hoc test Data are represented as means + SEM; *p < 0.05, **p < 0.01, ****p < 0.0001; Assays were performed in triplicate from 3 mice in each group.

To investigate whether genotype/sex has an effect on the myogenesis of PC satellite cells, mRNA levels were examined for Pax7, myogenin, embryonic myosin (MyHC3) and adult myosin (MyHC2) in PC muscle progenitors from both male and female wild-type and *mdx* mice at day 1, 3 and 7 of differentiation (Fig. 6A–D). In general Pax 7 levels decreased between 1 and 7 days of differentiation (p < 0.001) except in female *mdx* mice where they remained unaltered (Fig. 6A). Relative expression of Pax7 showed no genotype effect between wild-type and *mdx* except it was higher in male *mdx* on day-3. Although, there was no difference between male and female wild-type cells, Pax7 expression was 4-fold higher in male *mdx* compared to female *mdx* on day-3 (p < 0.01). Moreover, a 3-way ANOVA showed a significant interaction between mouse genotype and sex (p < 0.01).

Myogenin peaked on day-3 of differentiation for both sexes and genotypes (p < 0.01). Furthermore, myogenin in male *mdx* was 4.4 times the evident levels in female *mdx* on day 3 (Fig. 6B, p < 0.05). Embryonic myosin transcripts (MyHC3) were elevated on day-3 and day-7 compared to day-1 for both sexes and genotypes (Fig. 6C ANOVA, p < 0.01;). However, MyHC3 levels were generally lower in *mdx* females for all times points and a 3-way ANOVA indicated a significant interaction for sex and genotype (p < 0.05) despite post-hoc tests not revealing significance for a specific time point.

As expected, transcriptional level of adult myosin heavy chain (MyHC2) was most prevalent on day-7 in all groups. Noticeably, male *mdx* PC-derived myotubes exhibited 2.4 times the expression of MyHC2 than their wild-type counterparts (p < 0.0001; Fig. 6D). Moreover, male *mdx* cells demonstrated a 3.5-fold higher MyHC2 mRNA level compared to female *mdx* on day-7 (p < 0.0001; Fig. 6D).

### Immunodetection and quantification of satellite cells (SCs) in PC muscles from male and female wild-type and mdx mice

**Figure 7:**
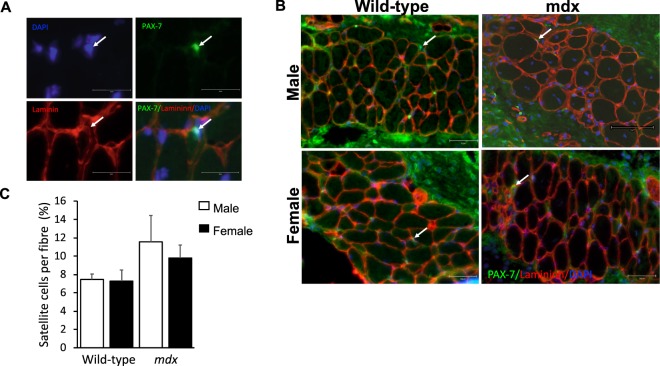
Satellite cells in PC muscle from different sex wild-type and *mdx* mice. Digitally magnified image showing representative satellite cell positive for Pax7 (green), colocalized with nuclear stain DAPI (blue) and located between the sarcolemma and the basal lamina delineated with laminin immunostaining (red). Arrows indicate location of SC. Scale bars = 25 μm. **(B)** PC fixed frozen cross-sections from 8-week old male wild-type and *mdx* mice, immunofluorescent stained with Pax 7 (green), laminin (red) and DAPI (blue). Images were captured using a 40x magnification lens. Arrows indicate example Pax7 positive SCs. All scale bars = 50 μm except male *mdx* = 75 μm. **(C)** Number of satellite cells per total number of muscle fibres expressed as a percentage quantified from immunofluorescent stained sections from male and female, wild-type and mdx mice (n = 4 mice for each group). Data were analysed by 2-way ANOVA and Tukey’s multiple comparison post-hoc test (P > 0.05). Data are represented as means + SEM; n = 4 mice.

Satellite cells (SCs) were identified by expression of Pax7 associated with nuclear staining and juxtaposed to the sarcolemma of muscle fibres as delineated by laminin immunostaining (Fig. 7A). Pax7 immunofluorescently stained PC muscle sections were quantified from both male and female wild-type and *mdx* mice (Fig. 7B,C). There were no significant differences between groups.

### Sex-related differences in the expression of genes encoding Ca^2+^-handling proteins in the PC from male and female *mdx* mice

**Figure 8:**
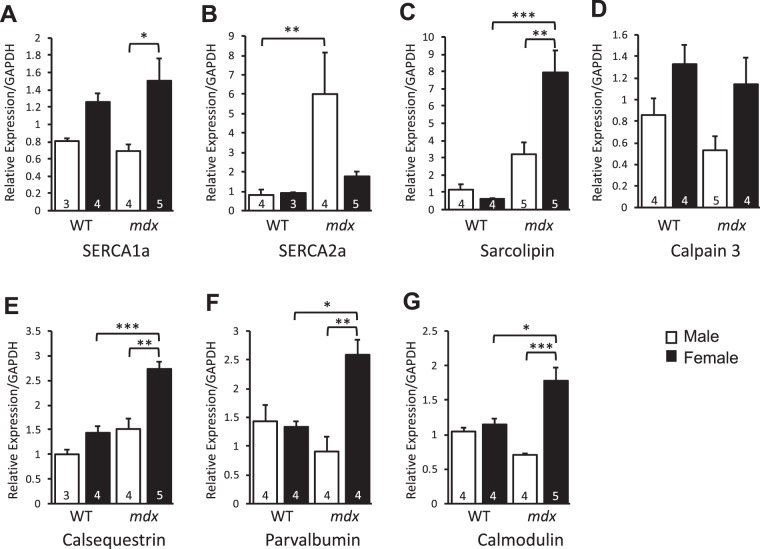
Relative expression of mRNA levels of genes encoding Ca^2+^-handling proteins in the PC from 8-week old male and female wild-type and *mdx* mice. The relative mRNA levels were determined by Taqman qPCR in the PC muscle tissue. Mean fold changes + SEM relative to a housekeeping gene GAPDH are represented from samples taken from 3–5 different mice (n size is indicated on each bar graph). Data were analysed by 2-way ANOVA and Tukey’s multiple comparison post-hoc test. *p < 0.05, **p < 0.01, ***p < 0.001. (n = 3–5).

The mRNA of five (Serca1a, Sln, Casq, Pvalb and Cam) of the seven transcripts quantified were expressed at significantly higher levels in PC from female *mdx* compared to the male *mdx* mice (Fig. 8). Although Serca2a mRNA was expressed at low absolute levels in all PC tissues, male *mdx* PC exhibited higher expression of Serca2a than wild-type male. No significant differences were seen between male and female levels for Capn3 mRNA in wild-type (p = 0.09) or *mdx* mice (p = 0.07). The comparison shows that the mRNA for five out of seven calcium regulatory proteins were expressed at higher levels in the PC of female compared to male *mdx* mice.

## Discussion

The regenerative capacity of different skeletal muscles is heterogeneous. This heterogeneity has been attributed to extrinsic and intrinsic differences including differences in the muscle specific stem cell populations^6,33^. There is an interest in understanding the variation in muscle regenerative capacity of particular muscles when injured or diseased as it may reveal mechanisms for therapy^34,35^. The dermal associated PC is a relatively unfamiliar striated muscle and although more commonly found in lower mammals; in humans, remnants of this tissue do exist as the platysma muscle of the neck, throat, palmaris brevis in the hand^36^ and the cremaster muscle in the scrotum^37^. The PC exhibits unique features including high levels of both remodelling and exogenous cell engraftment for reasons not clearly understood^25,28^. The PC also has a major advantage as being very accessible compared to other body musculature. The superficial location and thinness of the PC make it ideal for *in vivo* live imaging, which has been overlooked for studying muscle degenerative disorders^38^. In view of these features it was of interest to study the regeneration of PC in the *mdx* mouse model of DMD.

In present study we confirmed that the PC in healthy mice shows heterogeneity in fibre size and elevated central nucleated fibres (CNFs) in the absence of any damage, as previously reported^25^. These are two morphological features of myofibers undergoing remodelling or regeneration in postnatal skeletal muscle^39,40^. The relative high turnover in healthy PC was exacerbated in the *mdx* mouse, with both an increase in heterogeneity of fibre sizes and frequency of CNFs, typical of affected dystrophic muscles. There was a progressive increase in PC remodelling and regeneration with age, in both wild-type and dystrophic muscles respectively. The high myofiber turnover in the PC is not unique to this striated muscle as the craniofacial muscles including the masticatory muscles (MM), extraocular muscles (EOM)^41,42^ and laryngeal muscles (LM)^43^ have similar features that collectively set them apart from the majority of muscles (Review^44^). The craniofacial muscles in common with PC continually undergo myofiber remodelling^45^. The EOM has high regenerative capacity and remodeling activity, due to satellite cells with high proliferation potential and continual cell fusion respectively^34,46,47^. Additionally, the craniofacial muscles are spared from the severe dystrophic phenotype in DMD compared to other muscles. In contrast, our data revealed the PC was not spared in the *mdx* mouse.

The present study shows that the subdermal PC muscle is widely distributed in the mouse, with variable thickness which is comparable to earlier anatomical reports on PC in rodents and other animal species^48,49^. Noticeably, PC myofibers in *mdx* mice appeared hypertrophic and continued to exhibit a fast fibre phenotype compared to controls which is a phenotype reported in DMD mouse models^50^ and patients^51,52^.

Given the interest in sex differences in muscle regeneration and relevance to therapy we examined sexual dimorphism in the PC^15,53–55^. There was a two-fold greater level of CNFs in PC muscle from male compared to female *mdx* mice. However, these CNF differences may reflect increased muscle regeneration in males and/or decreased levels of degeneration in females. In support of muscle degeneration, it has been reported that female skeletal muscles of dystrophic mice and canines undergo less degeneration than their male counterparts^54,56^.

To gain an enhanced insight into the regenerative activity of PC, we chose to isolate the myogenic precursor cells in PC (satellite cells) from dystrophic mice. PC muscle precursors were examined *in vitro* when subjected to differentiation as previously undertaken on healthy mice^27,28^. Interestingly, in general we did not detect major differences in the expression of myogenic factors between wild-type and *mdx* muscle cells, nor between sex for wild-type mice, which is consistent with other reports in limb muscles^14^.

In the present study, we observed no difference in myogenic differentiation between sexes for normal healthy PC muscle progenitor cells *in vitro*, as reflected in myogenic factor gene expression profile over time from day 1 to day 7 of muscle differentiation. This is consistent with some reports for satellite cells in limb muscles^14^. However, PC cells from male *mdx* mice expressed differentiation genes earlier than female *mdx* mice, with elevated expression of myogenic genes Pax7 and myogenin in male compared to female *mdx* mice. Pax7 reflects a greater number of quiescent myogenic cells with self-renewal ability and elevated myogenin reflects increased myoblast fusion to differentiation. Adult myosin a marker of muscle maturation was more highly expressed in male *mdx* PC cells compared to female PC cells. These differences in myogenicity detected in cell culture may reflect intrinsic dimorphic differences between *mdx* male and female PC satellite cells respectively. We inferred myogenic activity from qPCR transcript levels of specific myogenic marker genes and not from protein as this had been previously validated by others for this assay^27,37^. Myogenin scoring was equally greater in both male wild-type and *mdx* mice compared to female counterparts. We are not sure the reasons for the appearance of a sex difference for myogenin protein staining in wild-type PC muscle and not from the myogenin qPCR assay. It is possible that the battery of qPCR genes is a more accurate assay for myogenicity compared to the relatively low level of detectable myogenin positive myo-nuclei in the differentiated PC cells in culture. We showed that SC densities in PC muscles were not different between muscle sexes nor genotypes. This further suggests that PC derived SCs from male mdx mice are intrinsically different to their female counterparts. We did not detect sex differences in myogenicity for limb muscle progenitor cells from *mdx* mice (not shown). We conclude that that sexual dimorphism in muscular dystrophic mice maybe another distinct response of the PC muscle compared with other muscles.

Some specialized skeletal muscles including extraocular, laryngeal and masticatory muscles are resistant to degeneration and this has been correlated with higher expression levels of calcium buffering and regulatory proteins^22,23,57^. Conversely, others have provided evidence that calcium handling is not related to muscle sparing of the extraocular muscles in *mdx* mice^35,58^. However, there is a consensus that calcium dysregulation contributes to the degeneration of muscle fibres in several muscular dystrophies^59–62^. In the present study, several genes regulating calcium homeostasis were significantly augmented in female versus male PC muscles from *mdx* mice. However, further examination is required to understand if these changes contribute to less regeneration/degeneration cycling in dystrophic female PC.

We concur that the PC is a fast-twitch muscle demonstrating high turnover in healthy muscle and augmented in a model of muscular dystrophy that further increases with age in mice. PC showed sexual dimorphism in muscular dystrophy with males having a higher muscle regenerative/degenerative activity than females. Sex determined intrinsic differences in the PC satellite cells derived from dystrophic mice included a differential propensity to differentiate and alterations in gene expression of Ca^2+^-handling proteins. PC strongly demonstrated the different phenotypical characteristics of muscular dystrophy and thereby validates this very accessible tissue for studying muscle degenerative disorders. This and other recent studies on PC set a premise to begin to utilize this muscle to expand our knowledge into the heterogeneity of muscle function and regeneration. In particular the use of *in vivo* imaging (e.g. intra-vital fluorescent microscopy) with fluorescent labelling and/or gene reporters should allow a clearer understanding of muscle regeneration *in vivo* using the PC as a novel model.

## Materials and Methods

### Animals

Control (C57BL/10ScSn) and *mdx* (C57BL/10ScSn-Dmd^mdx^/J) mice were obtained as breeding pairs from the Jackson Laboratory (Bar Harbor, ME, USA). Animals were housed in the Bio Resource Unit, Bioscience Research Building, NUIG, according to the institutional guidelines. Mice were maintained under controlled conditions (12:12 h light/dark cycle, 20–24 °C) and had access to water and food ad libitum. All experiments were performed and approved in accordance with the guidelines and regulations as defined by the NUIG Galway Animal Care Research Ethics Committee (ACREC) and under the authorization (AE19125/I075) issued by the Health Products Regulatory Authority (HPRA), Ireland.

### Tissue preparation

Dorsal skin tissues (1 cm^2^) and gastrocnemius muscles were harvested from mice after euthanization by CO_2_ asphyxiation. The dorsal skin was depilated by shaving and applying a depilatory cream. Tissues were placed on matching size of polyvinylidene difluoride (PVDF) membrane to maintain flat and immersed in 4% paraformaldehyde (PFA) at room temperature (RT) overnight followed by processing (Leica ASP300) and embedding in paraffin. Paraffin embedded tissues were transversely sectioned (5 μm) using a microtome (Leica Biosystems) and placed on glass slides for staining.

Immunofluorescent staining was performed on frozen sections following the preferred protocol for the antibodies used. Dermal tissues (~1 cm^2^) from the mid-dorsal region were harvested from 8-week-old male and female, wild type and *mdx* mice. Skin tissues were frozen in liquid-nitrogen-cooled isopentane (Merk), embedded in Tissue-Tek O.C.T. compound (Sakura) and stored at −80 °C. Frozen tissues were cryo-sectioned (10 μm) at −26 °C with a Leica CM1850 cryostat (Leica, Germany), placed on glass slides, and stored at −20 °C.

### Whole-body PC analysis

To analyse the distribution of PC muscle throughout the whole-body skin of the mouse, 1 cm^2^ sections of skin tissues from different anatomical locations were obtained from 1-year-old male wild-type and *mdx* mice. Transverse sections representative of each location was histologically stained with Trichrome blue kit (Abcam) following manufacturer’s instructions and digital images (10x magnified) captured under a bright-field microscope.

### Morphometric analysis

Paraffin sections were stained with Haematoxylin-Eosin (H&E) after processing them through deparaffinization and rehydration. Sections were then dehydrated and mounted with glass cover-slides using DPX mounting media (Sigma-Aldrich). Sections were stained with H&E following a standard approved protocol SOP ID number MDC1A_M.1.2.004, version 1: “*Histopathology in Hematoxylin & Eosin stained muscle sections*”.

Muscle regenerative capacity was assessed by quantifying the percentage of central nucleated muscle fibres (CNFs) on H&E stained wild-type and *mdx* tissue sections. Sections were imaged under bright-field microscopy using a 10x magnification lens. The proportion of CNFs was measured by analysing up to 1000 fibres per section taken from several non-overlapping images.

### Muscle immunofluorescent staining

Frozen cross-sections were equilibrated to RT, fixed in 4% PFA, permeabilized in 0.1% Triton-X100 in phosphate buffered saline (PBS), blocked in 5% goat serum followed by over-night incubation in the appropriate primary antibodies diluted in 1.5% goat serum at 4 °C in a humid environment.

Tissue genotype was confirmed by immunostaining with anti-dystrophin antibody. PC muscle fibre typing was performed by immunostaining serial sections with anti-fast and anti-slow myosin antibodies. To determine the fast isotype, unfixed frozen sections were double-stained with anti-MyHC-IIA and anti-MyHC-IIB to stain type IIA and type IIB myofibers respectively. The primary antibodies were revealed using an appropriate secondary antibody diluted in PBS.

Pax7 and Laminin double immunofluorescent staining was performed as follows. Frozen cross-sections were fixed in 2% paraformaldehyde (PFA), permeabilized in 0.3% Triton/0.1% Glycine, blocked in 3% bovine serum albumin with mouse-on-mouse (MOM) IgG blocking reagent (Vector Labs) followed by over-night incubation in primary antibodies mouse anti-Pax7 and rabbit anti-laminin diluted in 3% BSA/PBS at 4 °C. The primary antibodies were detected using the appropriate secondary antibody and then counterstained with DAPI. Slides were mounted and carefully covered with coverslips and allowed to dry overnight before visualisation under the EVOS M5000 Imaging System (ThermoFisher Scientific). Sections were visualised under GFP, RFP and DAPI channels, with individual and merged images taken for each channel. Non-overlapping microscopic images were taken from two muscle sections for each of four mice in each group. Satellite cells (SC) were determined as those that were co-stained for Pax7 and DAPI, and located along the basal lamina of muscle fibres as delineated with the laminin immunostaining. Analysis was carried out using a manual count method (multi-point tool) on ImageJ software (https://imagej.nih.gov/ij/), with %SCs calculated by number of SCs per total number of muscle fibres x 100.

### Muscle fibre sizing

Muscle fibre sizes were analysed on paraffin embedded transverse sections. Paraffin sections were pre-treated as described for immunofluorescence staining method for deparaffinization, dehydration and antigen retrieval. Sections were then treated by 0.1% Triton-X100/PBS for 5 min, washed and incubated with wheat germ agglutinin (WGA) conjugated to Alexa Fluor-488 (W11261; 1–10 µg/mL; ThermoFisher Scientific) for overnight at 4 °C. Images were taken at 20x magnification of 5 non-overlapping areas and analysed using ImageJ software (ImageJ, U. S. National Institutes of Health, Bethesda, Maryland, USA, https://imagej.nih.gov/ij/). Cross sectional area (CSA) and minimal Feret’s diameter (mFD) were measured as indices of muscle fibre size. Frequency distribution graphs were generated for CSA and mFD using bin sizes 100 µm^2^ and 5 µm respectively.

### Isolation, proliferation and striated muscle differentiation of dermal and gastrocnemius muscle precursor cells

Precursor cells of the PC muscle were isolated from the entire dorsal skin (superior to inferior) of 8-week-old wild-type and *mdx*, male and female mice as previously described^26,27^. A day prior to isolation, sterile 12 mm glass coverslips were coated with extracellular matrix (ECM). The ECM was prepared by incubating Cultrex basement membrane extract (2.77 mg/mL; Trevigen) and low molecular weight hyaluronan (2.5 mg/ml; R&D Systems) in pre-cooled phosphate-buffered saline (PBS; pH 7.4). PBS. ECM was applied to coverslips and allowed to polymerize at 37 °C for 24 hours in sterile conditions. Finally, excess PBS was eliminated and coverslips left to dry up for 1 h under laminar flow and UV light, prior to plating cells.

The entire dorsal skin of euthanized mice was shaven, washed in chlorhexidine and wiped with iodine. The skin was incubated for 30 min on ice in Hank’s balanced salt solution (HBSS; Gibco™), with 2% Fungizone™ (Gibco™) and 2% penicillin/streptomycin (P/S; Gibco).

Tissues were cut into pieces (2 mm^2^) and digested in collagenase type IA (2 mg/ml Sigma-Aldrich, C2674) at 37 °C for 2 hours shaking at 180 rpm. Undigested skin was mechanically dissociated by vigorous pipetting. The resulting cell suspension was filtered through a 40 µm cell strainer (BD), washed with proliferation medium containing Neurobasal A (Gibco™), 2% B27 (Gibco™), 1% L-glutamine 200 mM (Sigma-Aldrich) and 1% P/S and centrifuged at 1500 rpm for 5 min. Cells were resuspended in 5 ml of proliferation medium with 2% low serum growth supplement (LSGS; Gibco™), 40 ng/mL epidermal growth factor (EGF; R&D Systems), and 80 ng/mL basic fibroblast growth factor (FGF2; R&D Systems) and media was renewed every 2 days up to 7 days. Under these conditions the dermosphere-like structures formed within 48 h in culture (Suppl. Fig. 3).

For differentiation, dermospheres after 7 days of proliferation were gently disaggregated with 0.25% trypsin-EDTA solution (Sigma-Aldrich) until a mononucleated cell suspension was formed. Cells were resuspended in the differentiation medium (proliferation medium without growth factors plus 10% fetal bovine serum) before plating onto ECM-coated coverslips at a density of 75,000 cells/cm^2^ of each culture per coverslip. Cells were allowed to differentiate for different time points; 1, 3, and 7 days, and the medium was renewed every 2 days. At day 5–7 of differentiation, myoblast fusion and contractile activity were observed. Brightfield microscopic images of cultures as well as video recordings of twitching myotubes were taken by using a Nikon D90 digital camera coupled to a Nikon Eclipse TS100 microscope.

### Cell immunofluorescence and myogenesis assay

At the desired time point, cells were washed with PBS, fixed with 4% paraformaldehyde for 10 min at room temperature (RT), and further washed with PBS to wash off the fixative solution. After the cells were permeabilized and blocked in 5% goat serum diluted in 0.3% Triton X-100/PBS (PBST) for 1 hour at RT, the cells were incubated in primary antibodies/PBST for 2 hours at RT. Immunodetection of Pax7 represented the early stage of myogenesis (quiescent/activated satellite cell), myogenin represented the differentiation stage (differentiated myoblast fusion into myotubes) and adult Myosin heavy chain represented the generation of mature myotubes.

Primary antibodies were revealed using appropriate secondary antibodies. Nuclei were highlighted using either Hoechst (10 µg/mL) staining or ProLong^®^ Gold Antifade Mountant with DAPI (P36935; Molecular Probes™). Fluorescent images were captured using an upright fluorescent microscope (Olympus BX51, Olympus Corporation, Shinjuku, Tokyo, Japan) for tissue sections and Nikon Eclipse 80i microscope (Nikon Instruments Europe B.V., Amsterdam) for cell experiments and analysed by ImageJ software.

To assess myogenin expression, nine different fields from two different coverslips were analysed for myogenin (Myog) immunopositive staining. All myogenin positive nuclei were counted including mono or multi-nucleated myocytes and compared to total cell count derived from nuclei count. Data were analysed by comparing the mean percentages of +MyoG nuclei/total nuclei generated from four different biological replicates for each sample.

Antibodies list: Primary mouse antibodies used were anti-dystrophin (SC-15376; 1:50/100; Santa-Cruz); anti-myosin heavy chain fast fibers (MyHC-II) (M4276; 1:400; Sigma), anti-myosin heavy chain slow fibers (MyHC-I) (M8421; 1:4000; Sigma), anti-MyHC-IIA (SC_71;1:50; DSHB), anti-MyHC-IIB (BF-F3; 1:50;DSHB); anti-Pax7 (Pax7-c; 1:50/100; DSHB); anti-myogenin (F5D;1:50; DSHB); and pan-myosin heavy chain (A4.1025; 1:50; DSHB) or (M7523; 1:50/100; Sigma). Primary rabbit antibody used included anti-laminin (ab11575; 1:250;abcam). Secondary antibodies used were goat anti-mouse Alexa Fluor-546, and -488; and goat anti-rabbit Alexa Fluor-546; 1:800; Invitrogen).

### Gene expression

Total RNA was extracted from cells pooled from three coverslips for each condition at day 1,3 and 7, using the miRNeasy Mini kit (QIAGEN, 217004) and transcribed into cDNA with the High-Capacity cDNA Reverse Transcription Kit (Applied Biosystems) according to the manufacturer’s instructions. Quantitative real-time PCR (qRT-PCR) was conducted as previously described^27^ using 25–50 ng cDNA and TaqMan gene expression assays on the 7900HT Fast Real-Time PCR System (ThermoFisher Scientific) for cells or StepOnePlus Real-Time PCR Systems (Applied Biosystems) for tissues. TaqMan assays included Pax7, Myogenin (Myog), embryonic myosin heavy chain (MyHC3) and adult myosin heavy chain (MyHC2). C_T_ values were normalized to TATA-Box Binding Protein housekeeping gene (TBP). Analysis of the relative expression of the studied genes was carried out following the 2^−ΔΔCT^ method^63^.

Dorsal PC tissues were harvested from the inferior region of the mouse (2 × 1 cm^2^) and used for the RNA analysis. The subcutaneous layer was mechanically scraped off into an Eppendorf tube on ice. Samples were then lysed in 1 mL Trizol (Sigma) using a tissue homogenizer (TissueRuptor II, QIAGEN). Total RNA was extracted and qRT-PCR was performed as before. TaqMan gene expression assays were run on a StepOnePlus Real-Time PCR System (Applied Biosystems) to detect sarco/endoplasmic reticulum Ca^2+^-ATPase1a (SERCA1a), sarco/endoplasmic reticulum Ca^2+^-ATPase 2a (SERCA2a), sarcolipin (SLN), calsequestrin1 (CASQ1), calmodulin1 (Calm1), Parvalbumin (Parv) and calpain 3 (Suppl. Table S1). C_T_ values were normalized to glyceraldehyde 3-phosphate dehydrogenase (GAPDH) housekeeping gene and 2^−ΔΔCT^ method was followed for indicating the relative expression of genes of interests.

### Statistical analyses

Statistical analyses were carried out using Excel and GraphPad Prism software. A two-way analysis of variance (ANOVA) was applied where two factors (animal genotype vs. age/sex) were present in an experiment. A three-way ANOVA was performed when 3 factors (animal genotype vs. sex vs. time-point) were present in one experiment (Fig. 6A–D). Subsequently, pairwise multiple comparisons (Tukey’s post-hoc test) was used to assess the statistical significance for all data. Data are expressed as mean ± S.E.M., and differences were considered statistically significant at *p < 0.05, **p < 0.01, ***p < 0.001 and ****p < 0.0001.

## Supplementary information

Supplementary Information

Supplementary Movie S1

## References

[1] Guiraud S (2015). The Pathogenesis and Therapy of Muscular Dystrophies. Annu Rev Genomics Hum Genet.

[2] Chamberlain JR, Chamberlain JS (2017). Progress toward Gene Therapy for Duchenne Muscular Dystrophy. Mol Ther.

[3] Judge LM, Haraguchiln M, Chamberlain JS (2006). Dissecting the signaling and mechanical functions of the dystrophin-glycoprotein complex. J Cell Sci.

[4] Heslop L, Morgan JE, Partridge TA (2000). Evidence for a myogenic stem cell that is exhausted in dystrophic muscle. J Cell Sci.

[5] Chang NC, Chevalier FP, Rudnicki MA (2016). Satellite Cells in Muscular Dystrophy - Lost in Polarity. Trends Mol Med.

[6] McCullagh KJ, Perlingeiro RC (2015). Coaxing stem cells for skeletal muscle repair. Adv Drug Deliv Rev.

[7] Dumont NA (2015). Dystrophin expression in muscle stem cells regulates their polarity and asymmetric division. Nature Medicine.

[8] Bellinger AM (2009). Hypernitrosylated ryanodine receptor calcium release channels are leaky in dystrophic muscle. Nat Med.

[9] Millay DP (2009). Calcium influx is sufficient to induce muscular dystrophy through a TRPC-dependent mechanism. Proc Natl Acad Sci USA.

[10] Yin H, Price F, Rudnicki MA (2013). Satellite cells and the muscle stem cell niche. Physiol Rev.

[11] Seale P (2000). Pax7 is required for the specification of myogenic satellite cells. Cell.

[12] Megeney LA, Kablar B, Garrett K, Anderson JE, Rudnicki MA (1996). MyoD is required for myogenic stem cell function in adult skeletal muscle. Genes Dev.

[13] Renault V (2000). Skeletal muscle regeneration and the mitotic clock. Exp Gerontol.

[14] Neal A, Boldrin L, Morgan JE (2012). The satellite cell in male and female, developing and adult mouse muscle: distinct stem cells for growth and regeneration. PLoS One.

[15] Kim JH (2016). Sex hormones establish a reserve pool of adult muscle stem cells. Nat Cell Biol.

[16] Schiaffino S, Reggiani C (2011). Fiber types in mammalian skeletal muscles. Physiol Rev.

[17] Webster C, Silberstein L, Hays AP, Blau HM (1988). Fast muscle fibers are preferentially affected in Duchenne muscular dystrophy. Cell.

[18] Gehrig SM, Koopman R, Naim T, Tjoakarfa C, Lynch GS (2010). Making fast-twitch dystrophic muscles bigger protects them from contraction injury and attenuates the dystrophic pathology. Am J Pathol.

[19] Moens P, Baatsen PH, Marechal G (1993). Increased susceptibility of EDL muscles from mdx mice to damage induced by contractions with stretch. J Muscle Res Cell Motil.

[20] Randolph ME, Pavlath GK (2015). A muscle stem cell for every muscle: variability of satellite cell biology among different muscle groups. Front Aging Neurosci.

[21] Kaminski HJ, al-Hakim M, Leigh RJ, Katirji MB, Ruff RL (1992). Extraocular muscles are spared in advanced Duchenne dystrophy. Ann Neurol.

[22] Kunert-Keil CH (2014). Differential expression of genes involved in the calcium homeostasis in masticatory muscles of MDX mice. J Physiol Pharmacol.

[23] Zeiger U, Mitchell CH, Khurana TS (2010). Superior calcium homeostasis of extraocular muscles. Exp Eye Res.

[24] Naldaiz-Gastesi, N., Bahri, O. A., Lopez de Munain, A., McCullagh, K. J. A. & Izeta, A. The panniculus carnosus muscle: an evolutionary enigma at the intersection of distinct research fields. *J Anat*, **233**, 275–288, 10.1111/joa.12840 (2018). 10.1111/joa.12840 PMC6081499 29893024

[25] Brazelton TR, Nystrom M, Blau HM (2003). Significant differences among skeletal muscles in the incorporation of bone marrow-derived cells. Dev Biol.

[26] Garcia-Parra P (2012). Modeling neural differentiation on micropatterned substrates coated with neural matrix components. Front Cell Neurosci.

[27] Garcia-Parra P (2014). Murine muscle engineered from dermal precursors: an *in vitro* model for skeletal muscle generation, degeneration, and fatty infiltration. Tissue Eng Part C Methods.

[28] Naldaiz-Gastesi N (2016). Identification and Characterization of the Dermal Panniculus Carnosus Muscle Stem Cells. Stem Cell Reports.

[29] Anderson JE, Ovalle WK, Bressler BH (1987). Electron microscopic and autoradiographic characterization of hindlimb muscle regeneration in the mdx mouse. Anat Rec.

[30] Dangain J, Vrbova G (1984). Muscle development in mdx mutant mice. Muscle Nerve.

[31] DiMario JX, Uzman A, Strohman RC (1991). Fiber regeneration is not persistent in dystrophic (MDX) mouse skeletal muscle. Dev Biol.

[32] McDonald, A. A., Hebert, S. L., Kunz, M. D., Ralles, S. J. & McLoon, L. K. Disease course in mdx:utrophin+/− mice: comparison of three mouse models of Duchenne muscular dystrophy. *Physiol Rep* **3**, 10.14814/phy2.12391 (2015). 10.14814/phy2.12391 PMC4425985 25921779

[33] Pavlath, G. K. *et al*. Heterogeneity among muscle precursor cells in adult skeletal muscles with differing regenerative capacities. *Dev Dyn* **212**, 495–508, 10.1002/(SICI)1097-0177(199808)212:4<495::AID-AJA3>3.0.CO;2-C (1998). 10.1002/(SICI)1097-0177(199808)212:4<495::AID-AJA3>3.0.CO;2-C 9707323

[34] Stuelsatz P (2015). Extraocular muscle satellite cells are high performance myo-engines retaining efficient regenerative capacity in dystrophin deficiency. Dev Biol.

[35] Porter JD (2003). Constitutive properties, not molecular adaptations, mediate extraocular muscle sparing in dystrophic mdx mice. FASEB J.

[36] De la Cuadra-Blanco C, Peces-Pena MD, Carvallo-de Moraes LO, Herrera-Lara ME, Merida-Velasco JR (2013). Development of the platysma muscle and the superficial musculoaponeurotic system (human specimens at 8–17 weeks of development). ScientificWorldJournal.

[37] Naldaiz-Gastesi N (2019). Isolation and characterization of myogenic precursor cells from human cremaster muscle. Sci Rep.

[38] Laschke MW, Menger MD (2016). The dorsal skinfold chamber: A versatile tool for preclinical research in tissue engineering and regenerative medicine. Eur Cell Mater.

[39] Briguet A, Courdier-Fruh I, Foster M, Meier T, Magyar JP (2004). Histological parameters for the quantitative assessment of muscular dystrophy in the mdx-mouse. Neuromuscul Disord.

[40] Grounds MD, Radley HG, Lynch GS, Nagaraju K, De Luca A (2008). Towards developing standard operating procedures for pre-clinical testing in the mdx mouse model of Duchenne muscular dystrophy. Neurobiol Dis.

[41] McLoon LK, Wirtschafter JD (2002). Continuous myonuclear addition to single extraocular myofibers in uninjured adult rabbits. Muscle Nerve.

[42] McLoon LK, Rowe J, Wirtschafter J, McCormick KM (2004). Continuous myofiber remodeling in uninjured extraocular myofibers: myonuclear turnover and evidence for apoptosis. Muscle Nerve.

[43] Goding GS, Al-Sharif KI, McLoon LK (2005). Myonuclear addition to uninjured laryngeal myofibers in adult rabbits. Ann Otol Rhinol Laryngol.

[44] McLoon LK, Thorstenson KM, Solomon A, Lewis MP (2007). Myogenic precursor cells in craniofacial muscles. Oral Dis.

[45] Wirtschafter JD, Ferrington DA, McLoon LK (2004). Continuous remodeling of adult extraocular muscles as an explanation for selective craniofacial vulnerability in oculopharyngeal muscular dystrophy. J Neuroophthalmol.

[46] Pawlikowski B, Pulliam C, Betta ND, Kardon G, Olwin BB (2015). Pervasive satellite cell contribution to uninjured adult muscle fibers. Skelet Muscle.

[47] Keefe AC (2015). Muscle stem cells contribute to myofibres in sedentary adult mice. Nat Commun.

[48] Langworthy OR (1924). The panniculus carnosus in cat and dog and its genetical relation to the pectoral musculature. Journal of Mammalogy.

[49] Langworthy OR (1925). A morphological study of the panniculus carnosus and its genetical relationship to the pectoral musculature in rodents. American Journal of Anatomy.

[50] Duddy W (2015). Muscular dystrophy in the mdx mouse is a severe myopathy compounded by hypotrophy, hypertrophy and hyperplasia. Skelet Muscle.

[51] Cros D, Harnden P, Pellissier JF, Serratrice G (1989). Muscle hypertrophy in Duchenne muscular dystrophy. A pathological and morphometric study. J Neurol.

[52] Kornegay, J. N. *et al*. The paradox of muscle hypertrophy in muscular dystrophy. *Phys Med Rehabil Clin N Am* **23**, 149–172, xii, 10.1016/j.pmr.2011.11.014 (2012). 10.1016/j.pmr.2011.11.014 PMC5951392 22239881

[53] Deasy BM (2007). A role for cell sex in stem cell-mediated skeletal muscle regeneration: female cells have higher muscle regeneration efficiency. J Cell Biol.

[54] Salimena MC, Lagrota-Candido J, Quirico-Santos T (2004). Gender dimorphism influences extracellular matrix expression and regeneration of muscular tissue in mdx dystrophic mice. Histochem Cell Biol.

[55] La Colla A, Pronsato L, Milanesi L, Vasconsuelo A (2015). 17beta-Estradiol and testosterone in sarcopenia: Role of satellite cells. Ageing Res Rev.

[56] Valentine BA, Cooper BJ, de Lahunta A, O’Quinn R, Blue JT (1988). Canine X-linked muscular dystrophy. An animal model of Duchenne muscular dystrophy: clinical studies. J Neurol Sci.

[57] Ferretti, R., Marques, M. J., Khurana, T. S. & Santo Neto, H. Expression of calcium-buffering proteins in rat intrinsic laryngeal muscles. *Physiol Rep* **3**, 10.14814/phy2.12409 (2015). 10.14814/phy2.12409 PMC4510619 26109185

[58] Porter JD, Karathanasis P (1998). Extraocular muscle in merosin-deficient muscular dystrophy: cation homeostasis is maintained but is not mechanistic in muscle sparing. Cell Tissue Res.

[59] Burr AR, Molkentin JD (2015). Genetic evidence in the mouse solidifies the calcium hypothesis of myofiber death in muscular dystrophy. Cell Death Differ.

[60] Goonasekera SA (2011). Mitigation of muscular dystrophy in mice by SERCA overexpression in skeletal muscle. J Clin Invest.

[61] Gehrig SM (2012). Hsp72 preserves muscle function and slows progression of severe muscular dystrophy. Nature.

[62] Mazala DA (2015). SERCA1 overexpression minimizes skeletal muscle damage in dystrophic mouse models. Am J Physiol Cell Physiol.

[63] Livak, K. J. & Schmittgen, T. D. Analysis of Relative Gene Expression Data Using Real-Time Quantitative PCR and the 2^−ΔΔCT^ Method. *Methods* **25**(4), 402–408 (2001). 10.1006/meth.2001.1262 11846609

[64] Treuting, P. M., Dintzis, S. M. & Montine, K. S. *Comparative anatomy and histology: a mouse, rat, and human atlas*. Second edition, (Academic Press, 2018).

