# Supplementary material for: The panniculus carnosus muscle: A novel model of striated muscle regeneration that exhibits sex differences in the mdx mouse
Source: Sci Rep. 2019 Nov 4;9:15964. doi: 10.1038/s41598-019-52071-2 (PMC6828975; doi:10.1038/s41598-019-52071-2)
Supplement: Supplementary file 1 — Supplementary Information [file 41598_2019_52071_MOESM1_ESM.pdf]

SUPPLEMENTARY INFORMATION

MANUSCRIPT TITLE:

The *panniculus carnosus* muscle: A novel model of striated muscle regeneration that exhibits sex differences in the *mdx* mouse

AUTHORS: Ola A. Bahri, Neia Naldaiz-Gastesi, Donna C. Kennedy, Antony M. Wheatley, Ander Izeta and Karl J.A. McCullagh

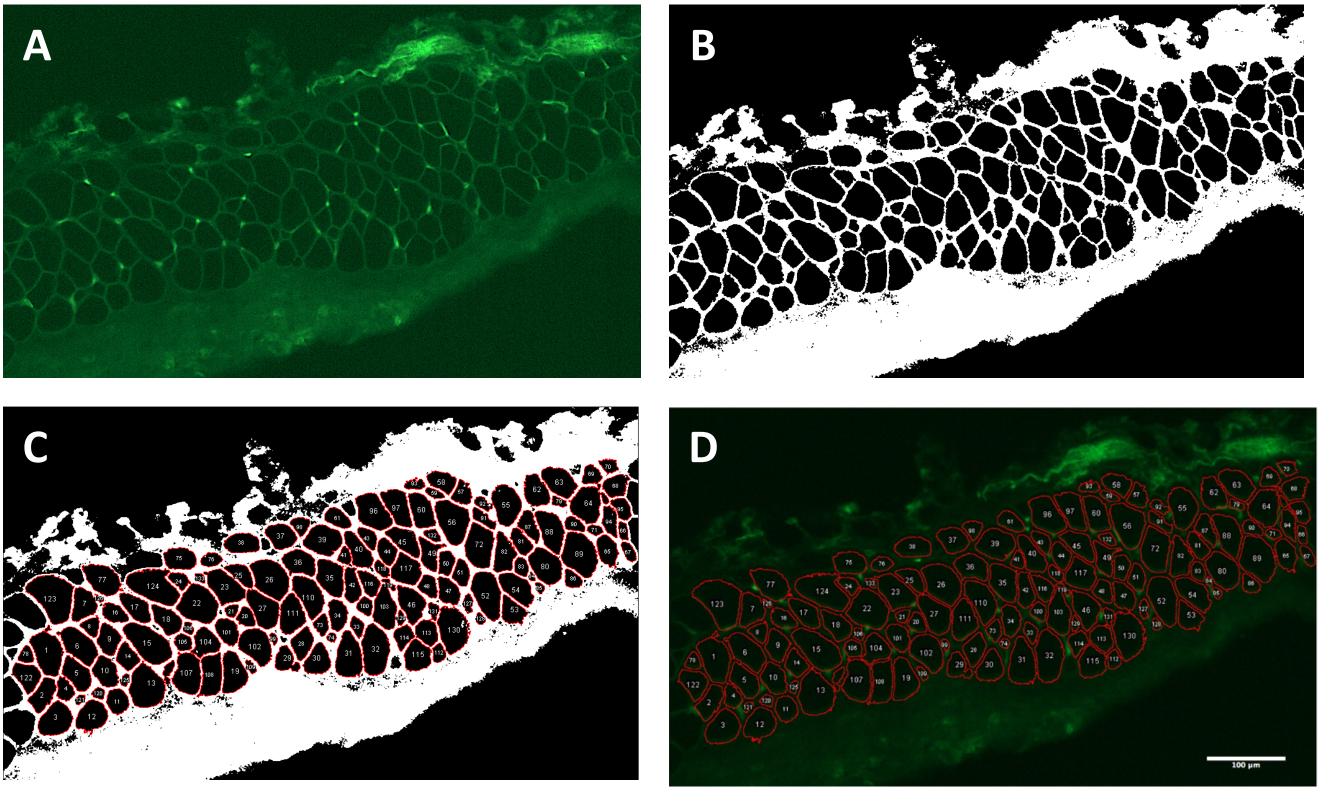

<u>Fig. S1.</u> **(A)** Representative image of PC section stained with wheat germ agglutinin (WGA) conjugated to Alexa Fluor-488 (green) highlighting the sarcolemma. (**B**) Converted imaged into binary by using ImageJ software. **(C)** Black inner area "muscle area" selected by the wand tool and ROI Manager tool in ImageJ software. (**D**) Numbered and measured myofibre area applied onto the original image. Scale bar= 100µm

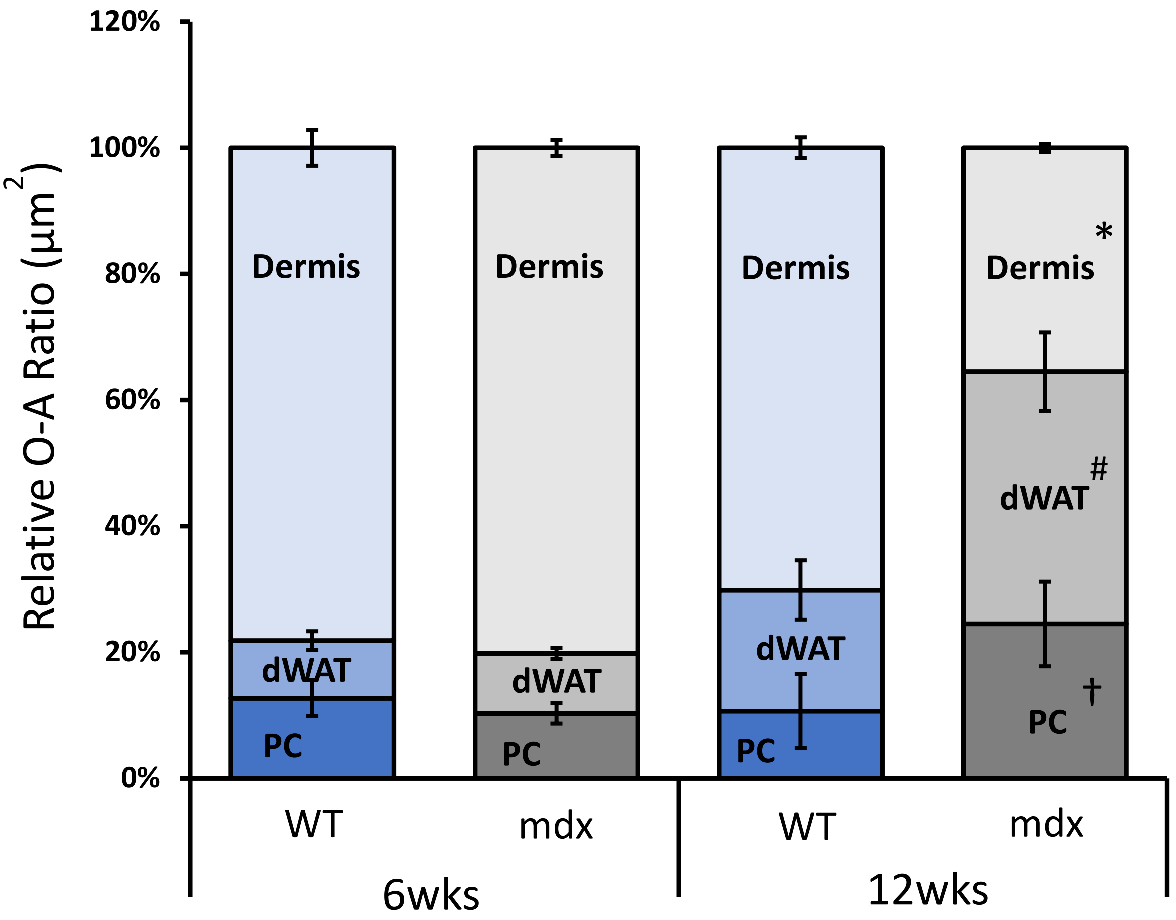

<u>Fig. S2.</u> Relative occupancy abundance (O-A) ratio of the different compartments of the dorsal skin: Dermis, dermal white adipose tissue (dWAT) and Panniculus Carnosus (PC). Data are represented as means $\pm$ SEM; n=4. Data were analysed by t-test: * Significant difference from WT, 12wks ($p<0.01$) and mdx 6wks($p<0.001$); # Significant difference from WT,12wks ($p<0.05$) and mdx 6wks ($p<0.01$); † Significant difference from WT,12wks ($p<0.001$) and mdx,6wks ($p<0.0001$).

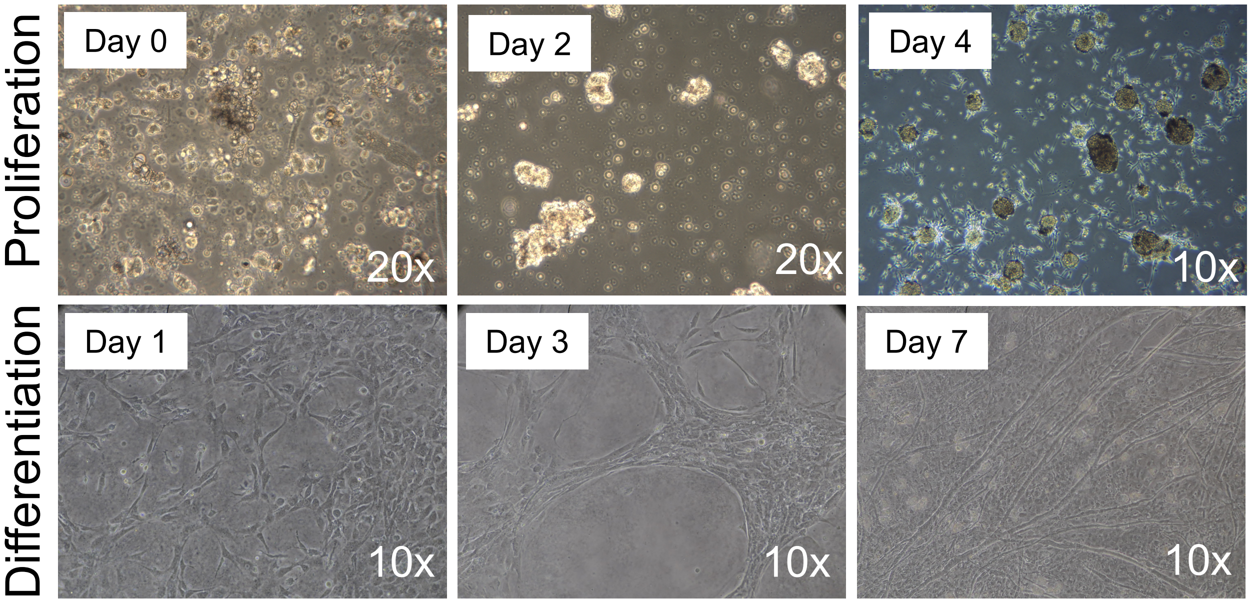

Fig. S3. Representative bright field microscopic images of dermal sphere cultures during proliferation from day 0 to day 4, and during differentiation day 1, 3, and 7. Notably, dermospheres were visible at day 2 and there were no muscle fragments by day 4 of proliferation.

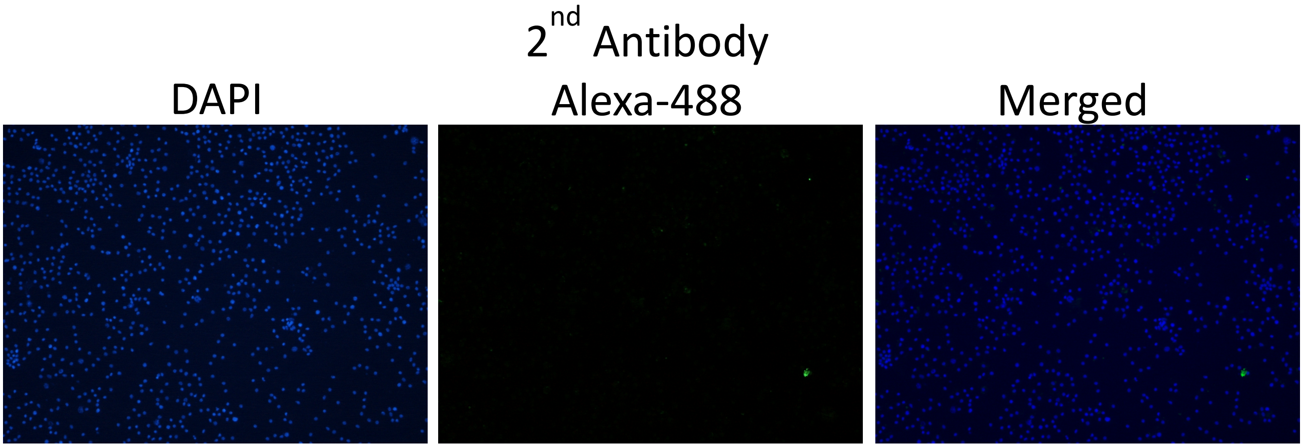

Fig. S4. Representative control immunofluorescent staining of differentiating muscle progenitor cells incubated with secondary antibody conjugated to Alexa-488 (Green). Nuclei are stained with DAPI (Blue). Images support that there was no background staining of muscle cells with the secondary antibody used in the immunofluorescent analyses.

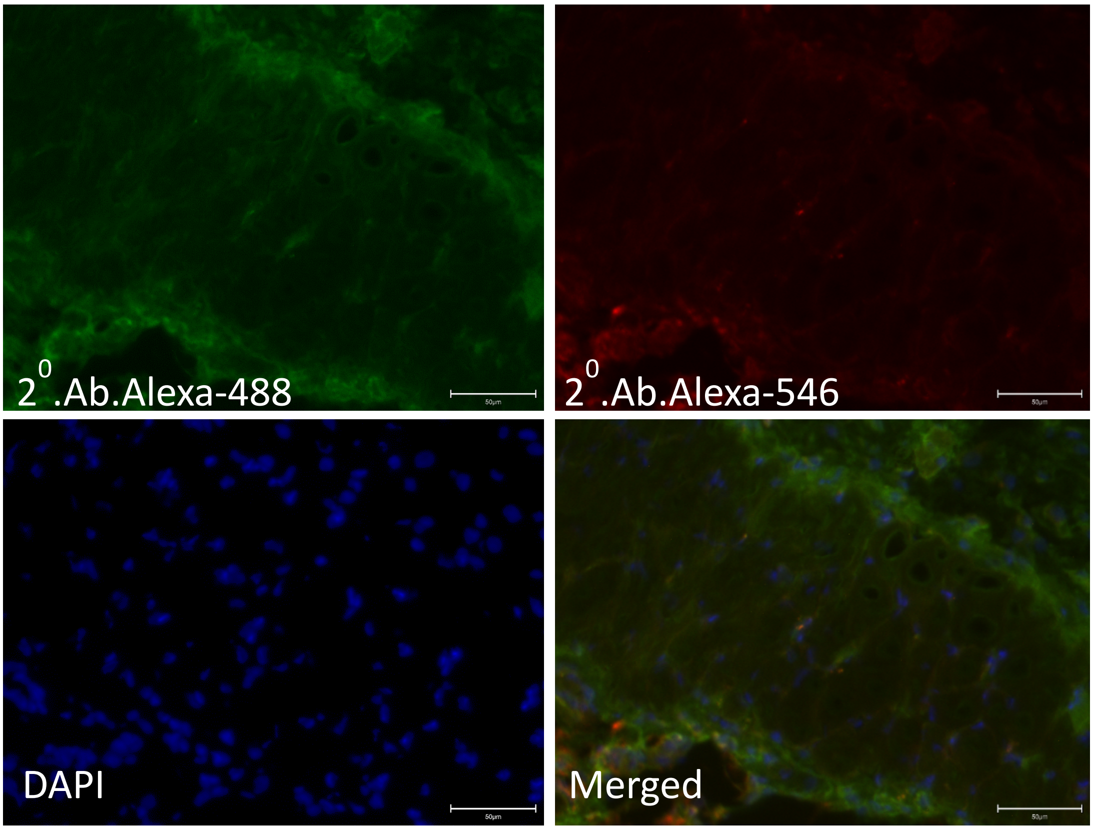

Fig. S5 Representative control immunofluorescent staining of PC muscle stained with secondary antibodies conjugated to Alexa-488 (Green) and Alexa-546 (Red) respectively. Nuclei are stained with DAPI (Blue). Images support that there was negative or negligible background staining of muscle cells with the secondary antibodies used in the immunofluorescent analyses.

Scale bar = 50μm

Movie S1: PC derived muscle progenitor cells from male and female wild-type and *mdx* mice differentiate into contractile myosin expressing myotubes by day 7 of differentiation.

Table S1:

Real-Time quantitative PCR assays: List of Taqman Primers

Gene	Symbol	Accession Number	Assay ID	Catalog No.	Company
Paired box protein 7	Pax7	NM_011039.2	Mm01354484-m1	4448892	ThermoFisher
Myogenin	Myog	NM_031189.2	Mm00446194_m1	4453320	ThermoFisher
Embyrionic Myosin Heahy Chain	eMyHC	NM_001099635.1	Mm01332463_m1	4448892	ThermoFisher
Adult Myosin Heavy Chain	Adult-MyHC	NM_001039545.2	Mm01332564_m1	4453320	ThermoFisher
Sarco/endoplasmic reticulum Ca^{2+}-ATPase 2a	SERCA1a	NM_007504(1)	Mm.PT.58.9990614	N/A	IDT
Sarco/endoplasmic reticulum Ca^{2+}-ATPase 1a	SERCA2a	NM_009722(3)	Mm.PT.58.5303089	N/A	IDT
Sarcolipin	SLN	NM_025540			IDT
Calpain3a	p94	NM_001109761.2			IDT
Calsequestrin	CASQ 1	NM_009813.2			IDT
Calmodulin	Calm	NM_001313934.1			IDT
TATA box binding protein	TBP	NM_013684.3	Mm00446973_m1	4453320	ThermoFisher
Glyceraldehyde 3-phosphate dehydrogenase	GAPDH	NM_008084(1)	Mm.PT.39a.1	N/A	IDT
