# Supplementary material for: The panniculus carnosus muscle: A novel model of striated muscle regeneration that exhibits sex differences in the mdx mouse
Source: Sci Rep. 2019 Nov 4;9:15964. doi: 10.1038/s41598-019-52071-2 (PMC6828975; doi:10.1038/s41598-019-52071-2)
Supplement: Supplementary file 2 — Supplementary Movie S1 [file 41598_2019_52071_MOESM2_ESM.zip › Supplementary Movie S1.pptx]

## Slide 1
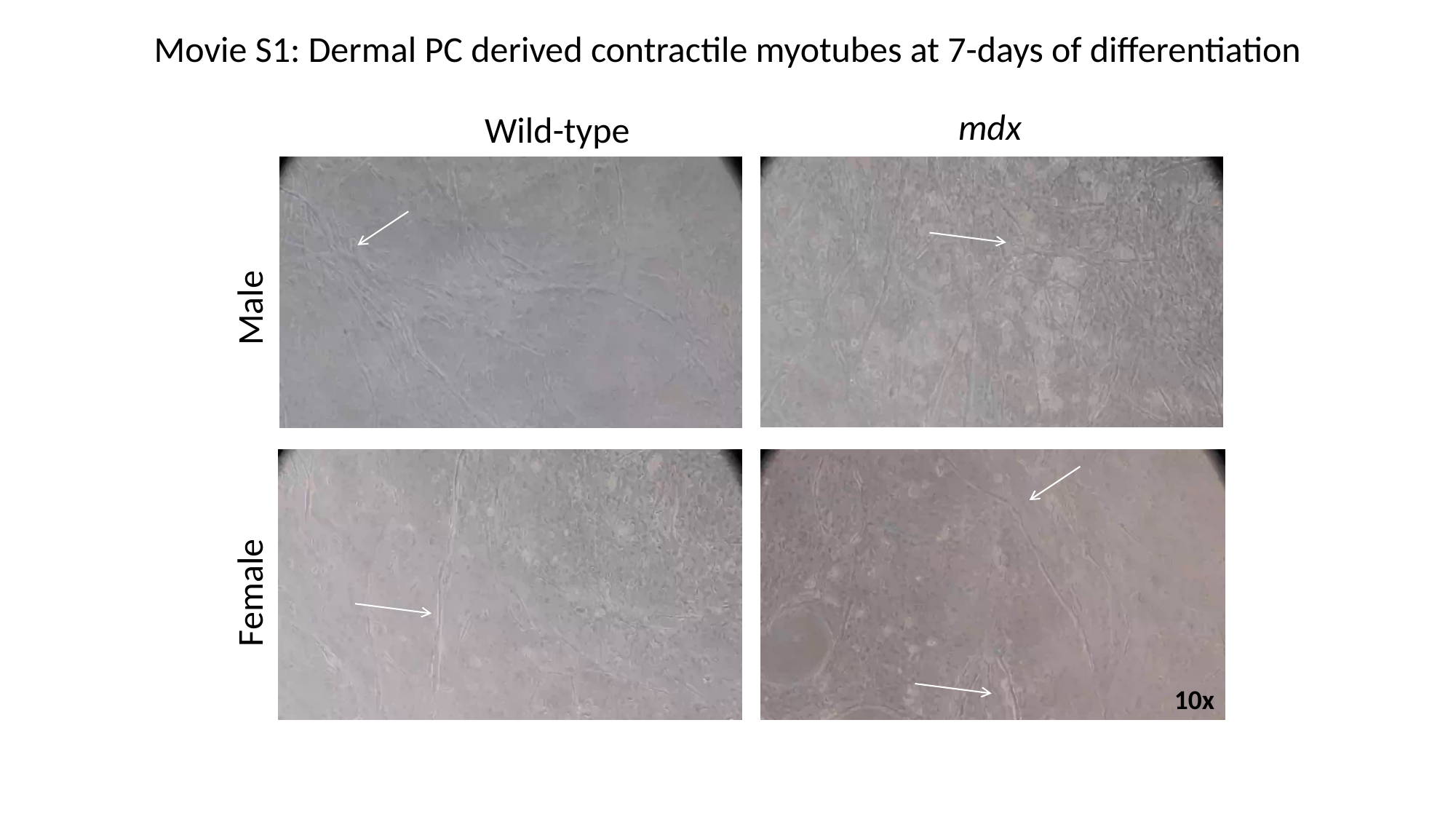

Movie S1: Dermal PC derived contractile myotubes at 7-days of differentiation
mdx
Wild-type
Male
Female
10x
